# A Life-Changing Moment in a Patient with Type 1 Diabetes: Insulin Holiday

**DOI:** 10.31661/gmj.v9i0.1769

**Published:** 2020-03-05

**Authors:** Muhammad Imran Butt, Noha Mukhtar, Muhammad Riazuddin, Lama Amer, Tarek Elsayed

**Affiliations:** ^1^King Faisal Specialist Hospital & Research Centre, Riyadh, Kingdom of Saudi Arabia; ^2^Al Faisal University, Riyadh, Kingdom of Saudi Arabia

**Keywords:** Autoimmunity, Insulin Secretion, Type 1 Diabetes, Autoimmune Diabetes, Islets of Langerhans, Diabetic Ketoacidosis

## Abstract

**Background:**

The diagnosis of type 1 diabetes can be a life-changing moment for our patients particularly having to live with the idea of taking insulin all their life and the barriers it can bring about.

**Case report:**

We report a patient with ten years of autoimmune type 1 diabetes treated with insulin. He was able to stop insulin in favour of oral diabetes agents after dynamic endocrine tests confirmed micro insulin secretion despite autoimmunity. Our case demonstrates that a detailed history and thorough investigations adopting a holistic approach can sometimes change the course of disease management and can leave a positive impact on the life of our patients.

**Conclusion:**

Patients with partial retention of beta-cell function in type 1 diabetes can temporarily stop insulin safely with ongoing surveillance.

## Introduction

The diagnosis and classification of diabetes is a straightforward process for most patients. However, in youth, this can be a challenge to classify diabetes. It is because type 1 diabetes can also present in middle age, although the peak age of presentation remains in the teenage. Type 2 diabetes presents in middle age, though, with increasing obesity, there is an increasing incidence at an earlier age [1]. The distinction between type 1 and type 2 diabetes is crucial as it helps understand the underlying pathophysiology and changes the treatment algorithm. A minority of the patients may instead have monogenic diabetes, which can be misdiagnosed and treated as either type 1 or type 2 diabetes. Identifying the aetiology of diabetes can direct specific treatment, tailored patient education, family screening, and surveillance.

## Case Presentation

We report a 27-year-old male who presented with a ten years’ history of Type 1 diabetes. He was keen to come off insulin to continue to work as a front line firefighter as an employer’s requirement. He was diagnosed when he presented to a remote hospital with a generalised illness, osmotic symptoms causing hospitalisation for a few days. He could not recollect the events and had no access to his medical records. He was free of complications of diabetes & was maintained on ten units BID of Lispro mix 50 insulin. He never had diabetic Ketoacidosis (DKA) and could miss insulin for 2-3 days with no adverse consequences. Occasionally, he would experience hypoglycemia after exertion. The maternal grandmother had type 2 diabetes treated with oral agents. His BMI was 24 kg/m2 with a bodyweight of 67 Kg. Glycemic control was excellent, as shown by HBA1c of 7.0%. To determine if the pancreatic endocrine function existed in our patient, we measured basal plasma insulin, c-peptide & glucose followed by a carbohydrate-rich meal. We then took serial measurements every 60 minutes up to three hours. We omitted insulin for 24 hours before and during the test. We used Roche E170 (Roche Diagnostics, Mannheim, Germany) Cobas analyser for measurement of glucose, Insulin and c-peptide levels. We used enzymatic reference method with hexokinase, third-generation for glucose and Electrochemiluminescence immunoassay, first-generation for both the insulin and c-peptide measurements. Results confirmed endogenous insulin secretion with a rise in plasma insulin and c-peptide levels in tandem with an increase in glucose levels (Table-1). This was in the setting of a weakly positive Glutamic acid decarboxylase antibodies (GADA) and Islet antigen-2 (IA-2A) and strongly positive Zn transporter 8 antibodies (ZnT8) confirming auto-immunity (Table-2). In the context of residual beta-cell function and maternal grandmother known with diabetes, we checked maternal HBA1c which was 7.4%.confirming diabetes With three generations in the family affected by diabetes in a person with residual Beta-cell function and no history of DKA, we performed maturity-onset diabetes of the young (MODY) screen which was negative( Table-3). We admitted him for a week to withhold insulin under direct observation for safety to decide if he can withdraw insulin, albeit temporarily. He remained well & we monitored capillary glucose, venous PH and urine ketones twice daily. He continued to be free of ketones with normal PH and with a maximal glucose excursion of 15 mmol/L. Our case serves a reminder that the proportion of patients with chronic autoimmune type 1 diabetes are micro secretors of insulin even after a decade of diagnosis which is sufficient to avoid lipolysis and ketosis but insulin & c- peptide levels are not at higher levels expected of patients with type 2 diabetes [2-4]. Although they have proven the existence of residual beta-cell function in type 1 diabetes, they have not commented if any patient was able to stop insulin. Our patient is managing diabetes without insulin with Sitagliptin 100mg daily since July 2018 with normoglycemia and without any episodes of DKA and HBA1c has improved to 6.4%. Mixed meal test 12 months after stopping insulin still shows micro insulin secretion with slightly lower levels of insulin which could be attributed to lesser glucose peak (Table-1).

## Discussion

Recent evidence shows that after a severe autoimmune insult, c- peptide levels decline during the initial seven years, followed by a time of continued stability [5]. This phenomenon looks possible in our patient who presented with a metabolic compromise coinciding with an autoimmune attack. Subsequent cooling of the immune process during its plateau phase might have protected some of his beta cells [5]. Our patient had a shorter duration of diabetes and onset at a later age, both of which are favourable predictors of residual beta-cell function [3,4]. He has remained free of complications of diabetes owing to the low level of preserved beta-cell activity, which curtails the complications of diabetes in particular severe hypoglycemic episodes & ketoacidosis [6]. Several histological studies have revealed healthy insulin-producing beta cells in patients with long-standing type 1 diabetes [2,7]. A conceivable explanation for this phenomenon of retention of beta-cell function could be attributed to “burnt out” autoimmunity, less severe autoimmunity or even beta cells being able to regenerate or circumvent immune attack. ZnT8 antibodies are more common in childhood diabetes and diminish and disappear earlier than GADA and IA-2A, which can remain positive for a lot longer (2, 4). Our patient, in contrast, presented during his late teens and retains ZnT8 antibodies with almost negative IA-2A antibodies for an obscure cause. We checked monogenic screen despite positive Islet autoantibodies as although rare, some studies have shown autoantibodies in MODY patients [8,9] and our patient had features to suspect MODY: fasting hyperglycemia, evidence of retained beta-cell function responsive to a mixed meal stimulation and three generations affected with diabetes. We chose Dipeptidyl peptidase 4 (DPP 4) inhibitor for his management as there is developing evidence that these agents have an immune modulatory effect leading to a rise in regulatory T cells including CD4+, CD25+ FoxP3+, reducing insulitis and pancreatitis [10]. It seems like a class effect. It involves beta-cell proliferation, increased beta-cell mass and enhanced insulin and c-peptide response to stimulation [11,12]. There have been some promising results with the use of DPP 4 inhibitors in clinical practice in patients with type 1 diabetes who have come off insulin in favour of DPP 4 inhibitors similar to our patient [13,14].

## Conclusion

 Patients with partial retention of beta-cell function in type 1 diabetes can temporarily stop insulin safely with ongoing surveillance.

## Acknowledgement

 None.

## Conflict of Interest

 None.

**Table 1:** Mixed Meal Test

	**Glucose mmol/L**		**C-peptide nmol/L**		**Insulin pmol /L ** **(17.8-173)**	
	Baseline	12 months later	Baseline	12 months later	Baseline	12 months later
**1 hour post meal**	9.0	10.3	0.101	0.075	244	154
**2 hour post meal**	13.2	10.1	0.125	0.076	334	155
**3 hour post meal**	13.6	8.9	0.113	<0.070	350	129

**Table 2:** Islet Autoantibodies Profile

	**Result**	**Reference**
**IA-2A (nmol/L)**	0.02	≤ 0.02
**ZnT8 U/ml**	206	<15.0
**GADA 65 (nmol/L)**	0.22	≤ 0.02

**IA-2A: **Insulinoma-Associated-2 Autoantibodies, **ZnT8: **Zinc transporter-8, **GADA:** Glutamic acid decarboxylase antibodies

**Table 3:** MODY Sequencing Panel

**Genes Analysed**	**Result**
ABCC8, APPL1, BLK, GCK, HNF1A, HNF1B, HNF4A, INS, KCNJ11, KLF11, NEUROD1, PAX4, PDX1	Negative

**MODY:** Maturity Onset Diabetes of the Young **ABCC8:** ATP-binding cassette, subfamily c, member 8 **APPL1: **Adaptor protein phosphotyrosine interaction, PH domain, and leucine zipper-containing protein 1 **BLK: **Tyrosine kinase, B lymphocyte-specific **GCK:** Glucokinase **HNF1A:** Hepatocyte nuclear factor 1-alpha **HNF1B: **Hepatonuclear factor 1-beta **HNF4A: **Hepatocyte nuclear factor 4-alpha **INS: **Insulin **KCNJ11: **Potassium channel, inwardly rectifying, subfamily j, member 11 **KLF11:** Kruppel-like factor 11 **NEUROD1: **Neurogenic differentiation 1 **PAX4: **Paired box gene-4 **PDX1:** Pancreas/duodenum homeobox protein 1
